# Prognostic Value of Plasma Epstein–Barr Virus DNA for Local and Regionally Advanced Nasopharyngeal Carcinoma Treated With Cisplatin-Based Concurrent Chemoradiotherapy in Intensity-Modulated Radiotherapy Era

**DOI:** 10.1097/MD.0000000000002642

**Published:** 2016-02-08

**Authors:** Wen-Hui Chen, Lin-Quan Tang, Shan-Shan Guo, Qiu-Yan Chen, Lu Zhang, Li-Ting Liu, Chao-Nan Qian, Xiang Guo, Dan Xie, Mu-Sheng Zeng, Hai-Qiang Mai

**Affiliations:** From the Sun Yat-sen University Cancer Center, State Key Laboratory of Oncology in South China, Collaborative Innovation Center for Cancer Medicine (W-HC, L-QT, S-SG, Q-YC, LZ, L-TL, C-NQ, XG, DX, M-SZ, H-QM); and Department of Nasopharyngeal Carcinoma, Sun Yat-sen University Cancer Center (L-QT, S-SG, Q-YC, LZ, L-TL, C-NQ, XG , H-QM), Guangzhou, PR China.

## Abstract

This study aimed to evaluate the prognostic value of plasma Epstein–Barr Virus DNA (EBV DNA) for local and regionally advanced nasopharyngeal carcinoma (NPC) patients treated with concurrent chemoradiotherapy in intensity-modulated radiotherapy (IMRT) era.

In this observational study, 404 nonmetastatic local and regionally advanced NPC patients treated with IMRT and cisplatin-based concurrent chemotherapy were recruited. Blood samples were collected before treatment for examination of plasma EBV DNA levels. We evaluated the association of pretreatment plasma EBV DNA levels with progression-free survival rate (PFS), distant metastasis-free survival rate (DMFS), and overall survival rate (OS).

Compared to patients with an EBV DNA level <4000 copies/mL, patients with an EBV DNA ≥4000 copies/mL had a lower rate of 3-year PFS (76%, 95% CI [68–84]) versus (93%, 95% CI [90–96], *P* < 0.001), DMFS (83%, 95% CI [76–89]) versus (97%, 95% CI [94–99], *P* < 0.001), and OS (85%, 95% CI [78–92]) versus (98%, 95% CI [95–100], *P* < 0.001). Multivariate analysis showed that pretreatment EBV DNA levels (HR = 3.324, 95% CI, 1.80–6.138, *P* < 0.001) and clinical stage (HR = 1.878, 95% CI, 1.036–3.404, *P* = 0.038) were the only independent factor associated with PFS, pretreatment EBV DNA level was the only significant factor to predict DMFS (HR = 6.292, 95% CI, 2.647–14.956, *P* < 0.001), and pretreatment EBV DNA levels (HR = 3.753, 95% CI, 1.701–8.284, *P* < 0.001) and clinical stage (HR = 2.577, 95% CI, 1.252–5.050, *P* = 0.010) were significantly associated with OS. In subgroup analysis, higher plasma EBV DNA levels still predicted a worse PFS, DMFS, and OS for the patients stage III or stage IVa-b, compared with those with low EBV DNA levels.

Elevated plasma EBV DNA was still effective prognostic biomarker for local and regionally advanced NPC patients treated with IMRT and cisplatin-based concurrent chemotherapy. Future ramdomized clinical trials are needed to further evaluate whether plasma EBV DNA levels could be applied to guide concurrent chemotherapy regimen for local and regionally advanced NPC patients.

## INTRODUCTION

Nasopharyngeal carcinoma (NPC) is an endemic in Southern China and Southeast Asia, with a peak incidence of 50 cases per 100,000.^1^ According to the data from GLOBOCAN on Cancer Research, there were >84,000 new NPC cases and 51,600 NPC-related deaths in 2012, with 80% of the cases located in Asia, especially China, and 5% in Europe and the USA.^2^ Radiotherapy is the primary treatment modality because of its high degree of radiosensitivity and inherent anatomic constraints, and concurrent chemoradiotherapy (CCRT) is currently considered as the standard treatment regimens of local and regionally advanced nasopharyngeal carcinoma.^3–6^ Recently, the quantification of pretreatment plasma Epstein–Barr virus (EBV) DNA was demonstrated a useful biomarker for the risk stratification, monitoring and prediction of the prognosis of NPC,^7–11^ but these findings were based on 2-dimensional radiotherapy (2D-CRT) or 3-dimensional radiotherapy (3D-CRT). Recently, compared with 2D-CRT, intensity-modulated radiotherapy (IMRT) has been demonstrated to gain superior locoregional control and improved long-term survival and was consider as the primary means of radiotherapy for NPC patients.^12,13^ The value of plasma EBV DNA for NPC patients treated with IMRT is still scarce. Chen^14^ have reported that the patients with a high pretreatment EBV DNA level had a worse disease-free survival (DFS) with short follow-up time, ∼2 years. Therefore, it is of interest to determine whether the plasma EBV DNA still has effective prognostic value, with a longer follow-up time, for local and regionally advanced patients treated with concurrent CCRT in IMRT era. Therefore, we conducted a retrospective cohort study to evaluate the prognostic role of plasma EBV DNA for local and regionally advanced NPC patients treated with IMRT and cisplatin-based concurrent chemotherapy.

## PATIENTS AND METHODS

Patients with biopsy-proven WHO type II or III and staged III–IVa-b locoregionally advanced nasopharyngeal carcinoma who were suitable for definitive chemoradiotherapy were eligible. In addition, patients were required to be >18 years old, with the value of Eastern Cooperative Oncology Group performance status (ECOG) of 0 or 1, and adequate hematologic, renal, and hepatic function. Patients were excluded if they are pregnant or lactating, or lost during the follow-up, or had a history of previous or synchronous malignant tumors, or previously received any anticancer therapy.

All patients were evaluated by complete physical examination, magnetic resonance imaging (MRI) of the head and neck, chest radiography, fiberoptic nasopharyngoscopy, abdominal sonography, and whole-body bone scan or^18^ FDG PET/CT. The following baseline data of sex, age, hereditary NPC, and smoking status was collected before treatment. The study was approved by Sun Yat-sen University's independent ethics committees and all patients provided written informed consent. According to the seventh American Joint Committee on Cancer (AJCC) TNM staging manual, all the patients were restaged, and finally 404 NPC patients treated with IMRT and cisplatin-based concurrent chemotherapy were consecutively recruited from January 2008 to December 2012.

### EBV DNA Measurement

As described in previous studies,^11,15–20^ patient plasma EBV DNA concentrations were measured by q-PCR before treatment. A cutoff level of 4000 copies/mL was chosen to define low and high EBV DNA levels because this threshold has previously been shown to be prognostic in previous NPC studies using the same measurement system.^7,9,20^ EBV-specific VCA/IgA antibodies and EBV-specific EA/IgA antibodies were measured using an immunoenzymic assay described previously.^21^ The cutoff value of VCA-IgA (≥1:80 vs <1:80) and EA-IgA (≥1:10 vs <1:10) was according to previous published literature.^18,22^

### Chemotherapy and Radiation Therapy

All patients underwent CCRT, consisting of concurrent cisplatin (30–40 mg/m^2^) weekly or (80–100 mg/m^2^) chemotherapy on day1, 22, and 43 during radiation therapy.^6,23^ A total of 141 patients (34.9%) received weekly chemotherapy, and 263 patients (65.1%) received chemotherapy 2 or 3 cycles every 3 weeks. The design of IMRT plan was based on previous studies.^12,24,25^ All patients were treated according to the principles of treatment for NPC patients at Sun Yat-sen University Cancer Center, Guangzhou, China.

### Outcome and Follow-Up

Our primary endpoint was progression-free survival (PFS), and distant metastasis-free survival (DMFS) and overall survival (OS) was included as secondary endpoints in this study. Progression-free survival was calculated from the date of initial treatment to the date of disease progression (local/regional recurrence or distant metastasis) or death from any cause or the censoring of the patient at the date of the last follow-up. DMFS was determined from the date of initial treatment to the date of distant relapse or death from any cause or patient censoring at the date of the last follow-up. OS was calculated from the date of initial treatment to the date of death from any cause or patient censoring at the date of the last follow-up. After treatment was completed, the patients were evaluated at 3-month intervals for the first 3 years and every 6 months thereafter. A complete physical examination and detailed history were performed at the time of each follow-up visits, and plasma EBV DNA, routine blood and biochemistry tests were carried out by collecting peripheral blood. MRI of the head and neck, nasopharyngescopy, chestradiography, abdominal sonography, a whole-body bone scan or PET/CT were routinely performed annually or at the time of the clinical suggestion of tumor recurrence.

### Statistical Analysis

A Mann–Whitney test was used to detect differences of plasma EBV DNA for the subgroup patients with or without clinical events (progression, distant metastasis, or death). The Kaplan–Meier method was used to estimate the cumulative survival plot (<4000 or ≥4000 copies/mL) and compared using the log-rank test. Univariate analysis was performed for each of the variables, and variables with *P* value ≤0.05 were subjected to multivariate analysis. Multivariate analysis was performed using a Cox proportional hazards model, excluding insignificant variables by backward elimination. Factors were included in univariate analysis as follow: age (>45 years vs ≤45 years), sex (male vs female), clinical stage (IVa-b vs III), smoking status (yes vs no), family history of NPC (yes vs no), EBV DNA (≥4000 copies/mL vs <4000 copies/mL), VCA-IgA (≥1:80 vs <1:80), and EA-IgA (≥1:10 vs <1:10). All reported probability values were 2 tailed, and *P* < 0.05 was considered statistically significant. All statistical analyses were performed using SPSS 17.0 (SPSS Inc., Chicago, IL).

## RESULTS

### Patient Characteristics and Distribution of Plasma EBV DNA Level in the Study Population

**FIGURE 1:**
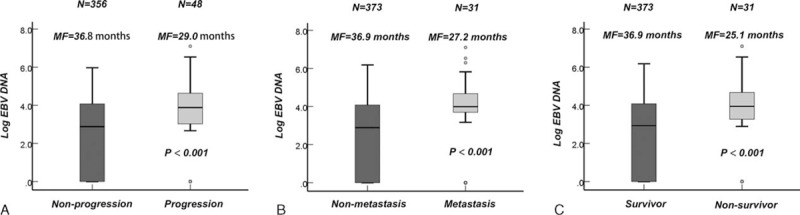
Log (EBV DNA) are expressed as the median and 5% to 95% percentile in patients (A) with/without progression, (B) with/without distant metastasis, and (C) survivor or deaths. *P* value was calculated by Wilcoxon rank-sum test.EBV DNA = Epstein–Barr Virus DNA, MF = median follow-up time.

The pretreatment characteristics of NPC patients are listed in Table 1. The median follow-up time was 33.5 months (IQR: 24.2–51.8). Forty-eight patients emerged disease progression, 31 patients developed distant metastases (DM), and 31 patients dead at the date of the last follow-up. Patients who have disease progression had significantly higher EBV DNA levels before treatment than patients without disease progression during follow-up of this study: 7645 copies/mL (25th–75th percentile: 947–45,550 copies/mL) and 760.1 copies/mL (25th–75th percentile: 0–11,825 copies/mL), respectively (Figure 1A, *P* < 0.001). The median level of plasma EBV DNA was higher in patients with DM compared to patients without DM (Figure 1B, P <0.001), with median values of 9870 copies/mL (25th–75th percentile: 4360–57,800 copies/mL) and 776.9 copies/mL (25th–75th percentile: 0–12,500 copies/mL), respectively. Patients who was dead had significantly higher pretreatment EBV DNA levels than patients still alive during the follow-up time: 8840 copies/mL (25th–75th percentile: 947–45,550 copies/mL) and 856.4 copies/mL (25th–75th percentile: 0–11,825 copies/mL), respectively (Figure 1C, *P* <0.001).

**TABLE 1:**
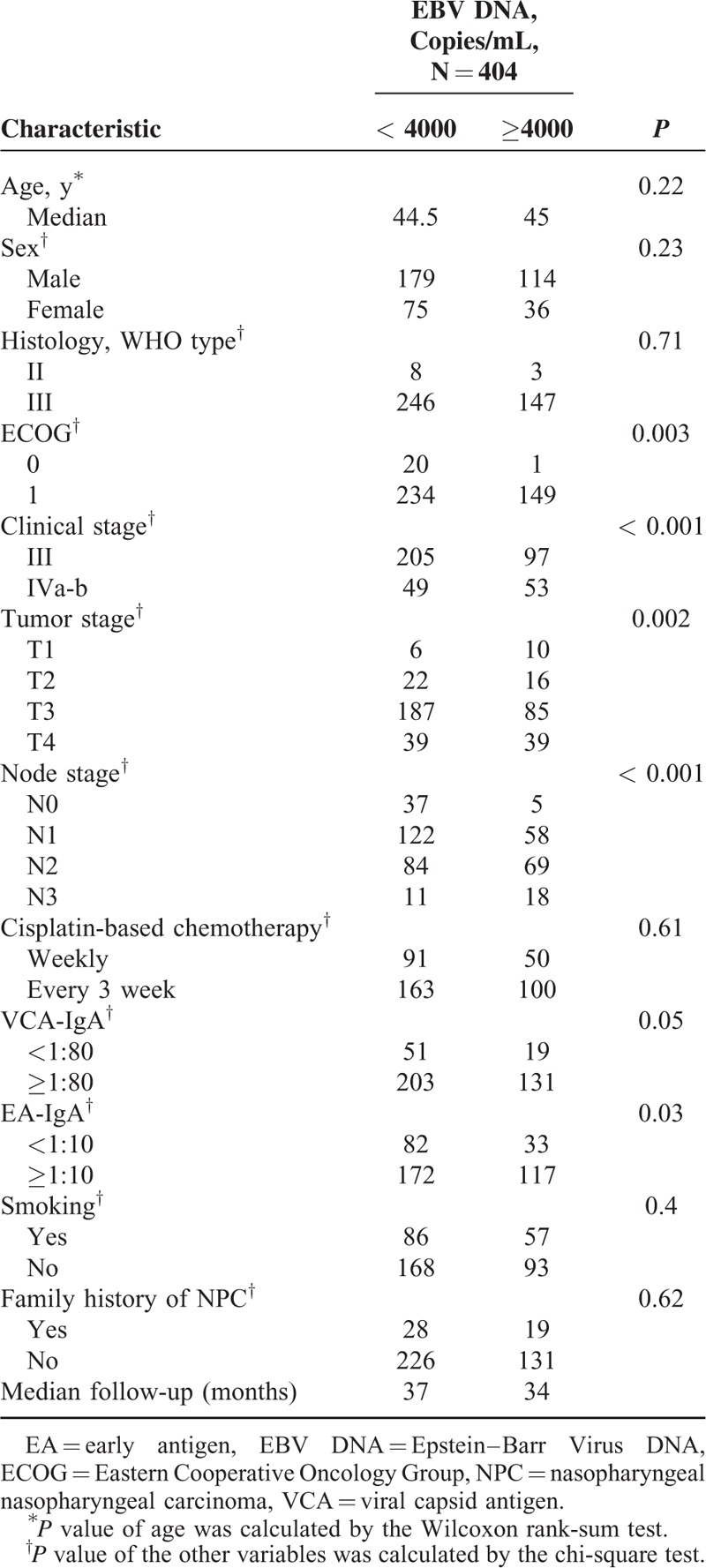
Patient Demographics and Clinical Characteristics

The plasma EBV DNA level was higher in patients staged IVa-b than in patients with staged III (*P* < 0.001), with median values of 5105 copies/mL (25th–75th percentile: 0–38, 850 copies/mL) and 620.6 copies/mL (25th–75th percentile: 0–8505 copies/mL), respectively. A correlation analysis further proved that plasma EBV DNA levels correlated with ECOG performance, T stage, N stage, tumor TNM stage, and EA-IgA (Table 1).

### Plasma EBV DNA Association With PFS, DMFS, and OS, and Multivariate Analyses of Pretreatment EBV DNA Levels as the Prognostic Factor

**FIGURE 2:**
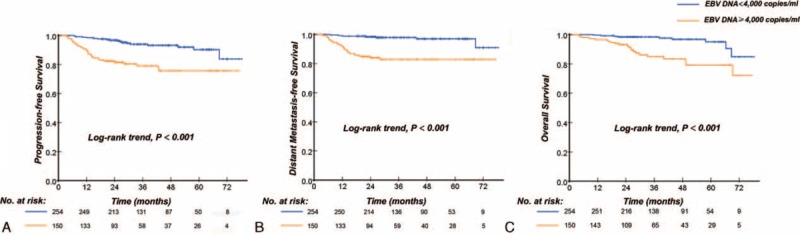
Kaplan–Meier curves of progression-free survival, distant metastasis-free survival, and overall survival according to the pretreatment EBV DNA levels (<4000 copies/mL vs ≥4000 copies/mL) for local and regionally advanced NPC patients: disease-free survival (A), distant metastasis-free survival (B), and overall survival (OS).EBV DNA = Epstein–Barr Virus DNA, NPC = nasopharyngeal carcinoma, OS = overall survival.

Compared to patients with a low pretreatment EBV DNA level, patients with a higher EBV DNA level had a lower rate 3-year of PFS, (76%, 95% CI [68–84]) versus (93%, 95% CI [90–96], *P* < 0.001, Figure 2A), DMFS (83%, 95% CI [76–89]) versus (97%, 95% CI [94–99], *P* < 0.001, Figure 2B), and OS (85%, 95% CI [78–92]) versus (98%, 95% CI [95–100], *P* < 0.001, Figure 2C). Univariate analysis indicated that age, clinical stage, and plasma EBV DNA was associated with treatment failure of NPC (Table 2).

**TABLE 2:**
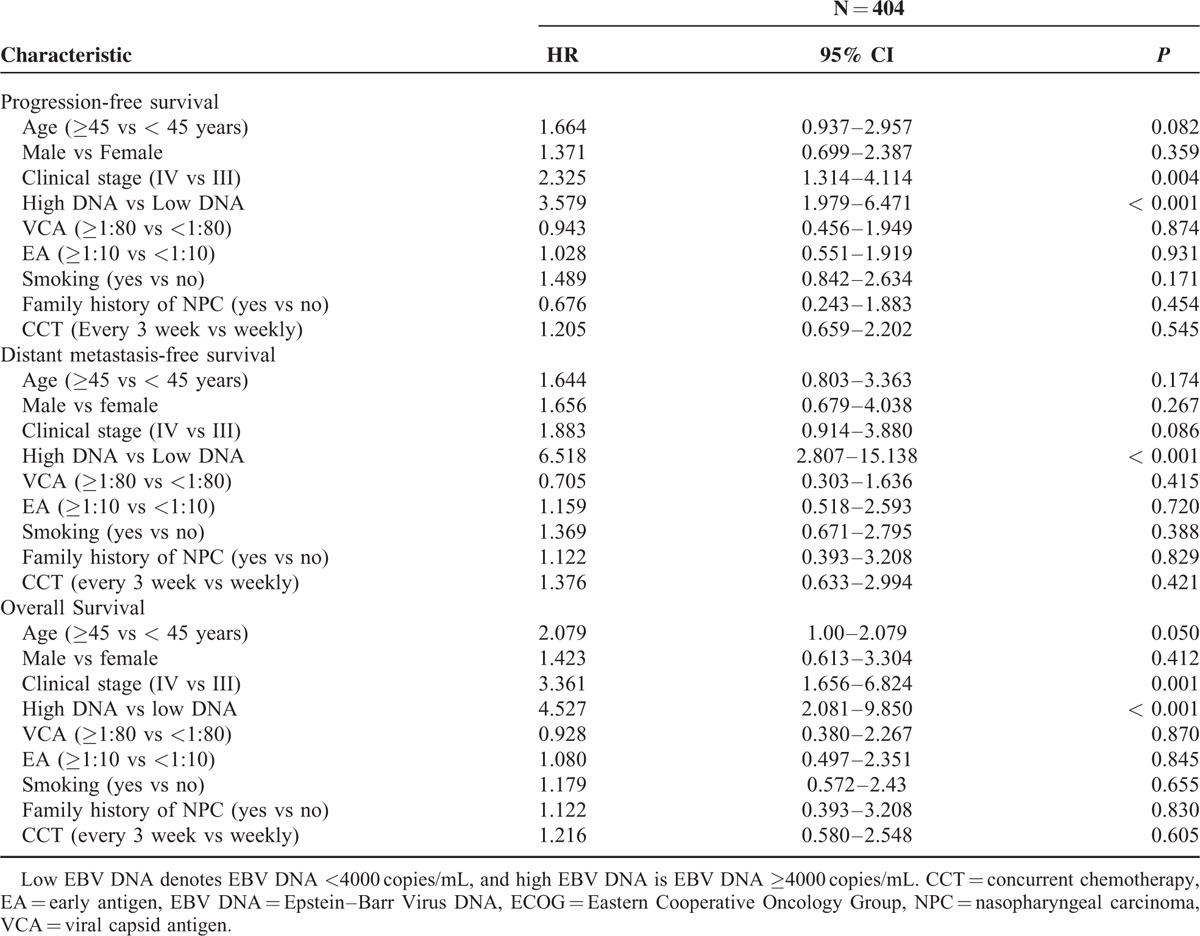
Univariate Cox Proportional Hazards Analysis

A multivariate Cox proportional-hazards model was generated that including the following variables: age, clinical stage, and plasma EBV DNA. The multivariate Cox regression analysis showed that the pretreatment EBV DNA levels (HR = 3.237, 95% CI, 1.775–5.901, *P* < 0.001) and clinical stage (HR = 1.901, 95% CI, 1.066–3.391, *P* = 0.029) were the only independent prognostic factor associated with PFS, pretreatment EBV DNA levels were the only significant factor to predict DMFS (HR = 6.518, 95% CI, 2.807 to 15.138, *P* < 0.001), and the pretreatment EBV DNA levels (HR = 3.753, 95% CI, 1.701–8.284, *P* < 0.001) and clinical stage (HR = 2.577, 95% CI, 1.252–5.050, *P* = 0.010) were significantly associated with OS (Table 3).

**TABLE 3:**
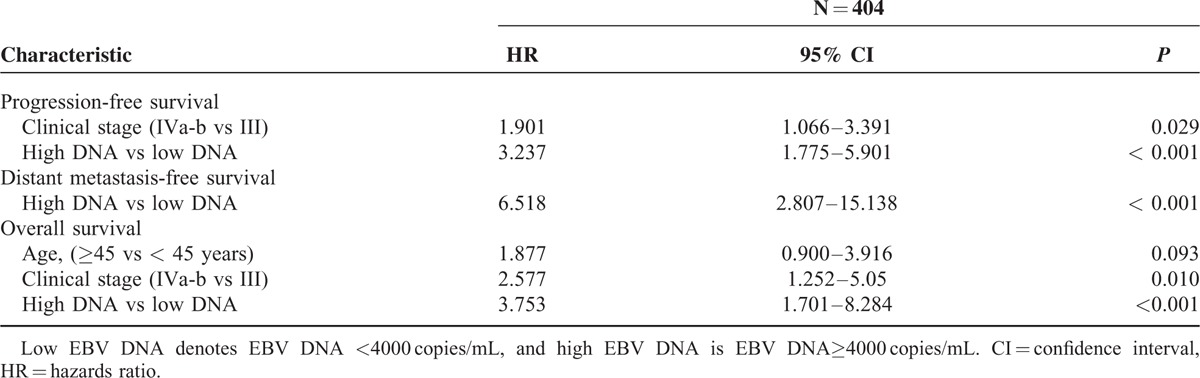
Multivariate Cox Proportional Hazards Analysis

### Prognostic Significance of EBV DNA Level Within the Stage III and Stage IVa-b Classification

**FIGURE 3:**
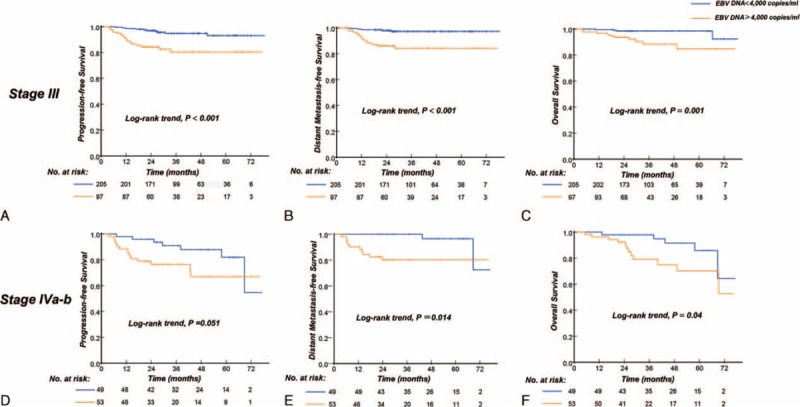
Kaplan–Meier curves of progression-free survival and distant metastasis-free survival according to the pretreatment EBV DNA levels (<4000 copies/mL vs ≥4000 copies/mL) for subgroup analysis. Progression-free survival (A), distant metastasis-free survival (B), and overall survival (C) for patients staged III; progression-free survival (D), distant metastasis-free survival (E), and overall survival (F) for patients staged IVa-b. EBV DNA = Epstein–Barr Virus DNA.

Given the independent prognostic significance of elevated EBV DNA levels in local and regionally advanced NPC patients, we evaluated the discrimination power of elevated EBV DNA levels in **s**tage III and stage IVa-b patients. In subgroup analysis, for stage III patients, compared to patients with low EBV DNA levels, patients with elevated EBV DNA levels had lower rate 3 year of PFS (81%, 95% CI [72–89]) versus (95%, 95% CI [92–98], *P* < 0.001), DMFS (86%, 95% CI [79–93]) versus (97%, 95% CI [95–100], *P* < 0.001), and OS (88%, 95% CI [81–96]) versus (98%, 95% CI [97–100], *P* = 0.001) (Figure 3A–C). For staged IVa-b patients, the patients with high EBV DNA levels compared with those displaying low pretreatment EBV DNA levels, the 3-year PFS was 73% (95% CI, 61%–85%) and 88% (95% CI, 78%–98%), respectively (*P* = 0.051), the 3-year DMFS was 80% (95% CI, 69%–91%) and 100%, respectively (*P* = 0.014), and the 3-year OS was 79% (95% CI, 67%–92%) and 95% (95% CI, 88%–100%), respectively (*P* = 0.04) (Figure 3D-F).

## DISCUSSION

Previous studies,^7,9–11,16^ based on 2D-CRT or 3D-CRT, have examined the association between EBV DNA and NPC prognosis. Even in IMRT era, Chen^14^ also reported that the 2-year disease-free survival (DFS) rates in patients with positive and negative pretreatment plasma EBV-DNA were 94.4% and 80.8%, respectively. In this study we have confirmed that plasma EB DNA is still an effective biomarker for predicting disease progression and distant metastasis for advanced NPC patients treated with IMRT and concurrent chemotherapy with much longer follow-up time. The results of this study were similar to the report by Chen.^14^ In recent year, intensity-modulated radiotherapy has been proven to be effective in increasing the local control rate for NPC patients; however, distant metastasis was still the main treatment failure and become the greatest challenge for NPC patients.^26^ Currently, the tumor-node metastases (TNM) staging system has been widely used to predict and guide the treatment regimen of NPC patients. According to the NCCN guideline, all the local and regionally advanced NPC patients in this study were received concurrent chemoradiothrapy with similar regimen and radiation technique. However, we found that even these patients received similar treatment regimen, patients with ≥4000 copies/mL of EBV DNA display a >6-fold increased risk of distant metastasis compared with patients with <4000 copies/mL of EBV DNA. This indicated that it is not enough effective to used single drug of cisplatin for the high-risk local advance NPC patients to reduced distant metastasis, although cisplatin-based concurrent chemoradiotherapy was considered as the standard regimen for advanced NPC patients. Our result have demonstrated that pretreatment EBV DNA levels was the only effective factor to predict DMFS, regardless of patients staged III or stage IVa-b. The patients with EBV DNA ≥4000 copies/mL took a higher rate of 10% to develop distant metastasis, compared with those of EBV DNA <4000 copies/mL. Therefore, the high-risk group of advanced NPC patients, stratified by plasma EBVDNA, should require more intense treatment.

Recent studies have demonstrated that the adjuvant chemotherapy (ACT) did not provided any benefit for local advance stage NPC patients^3,5,6^ and the effect of NACT on NPC remains controversial. Thus, we assumed that cisplatin-based concurrent chemotherapy plus other drug such as paclitaxel, docetaxel, or gemcitabine may reduce micrometastasis lesion for high-risk advanced NPC patients. One prospective, ramdomized phase III clinical is going to be conducted to assess the benefit of concurrent chemotherapy regimen of cisplatin plus paclitaxel compared with cisplatin alone in high-risk local and regionally advanced NPC patients, identified with pretreatment plasma EBV DNA ≥4000 copies/mL.

For local and regionally advanced NPC patients with EBV DNA <4000 copies/mL, regardless of in the entire population or patients staged III or IV, the 3-year's PFS and DMFS was approximate to 90%, according to the founding of this study. The National Comprehensive Cancer Network (NCCN) recommends that CCRT with cisplatin (CDDP) be delivered at a high dose at 3-week intervals or at an intermediate dose weekly for stage II–IVB NPC. Many previous published literatures,^27–30^ such as Intergroup 0099 (INT-0099),^27^ NPC-9901,^28^ and NPC-9902 trial,^29^ have demonstrated the local and regionally advanced NPC patients benefit from cisplatin-based concurrent chemotherapy. However, the proportion of patients in the chemoradiation arm completed all 3 cycles of concurrent chemotherapy was 52% to 63% because of chemotherapy-induced toxicity. Therefore, avoidance of concurrent chemotherapy-related toxicity effects and unnecessary cost is important to improve long-term outcomes for advanced NPC patients that have excellent prognosis. Moreover, the results of combining analysis of the NPC-9901 and the NPC-9902 Trials,^31^ reported by Lee, proved that the 5-year local and regionally failure-free rate (FFR), for patients who received 0–1, 2, and 3 concurrent cycles were 79%, 88%, and 88%, and respectively; the corresponding distant-FFR by adjuvant cycles were 68%, 78%, and 77%, respectively. The difference of survival curve between 2 and 3 cycles was insignificant. Lee^31^ and Chan^32^ also considered that the Intergroup-0099 regimen could be refined by reducing from 3 to 2 concurrent cycles (ie, reducing the dose of concurrent cisplatin to 200 mg/m^2^) without affecting the efficacy. In addition, intensity-modulated radiotherapy (IMRT), with 5-year disease-specific survival (DSS) ∼85%, has gradually replaced 2-dimensional conventional radiotherapy as the primary radiotherapy modality for the treatment of NPC, due to its superior local and regionally control and improve the long-term survival of NPC patients.^33,34^ Given the reason mentioned above, plasma EBV DNA levels might be applied to guide concurrent chemotherapy regimen for local and regionally advanced NPC patients. Therefore, we make a hypothesis that the low-risk locoregionally advanced NPC patients received 2 cycles of chemotherapy may gain similar long-term survival as those received 3 cycles of chemotherapy, leading to less chemotherapy-induced toxicity. One prospective, phase II noninferiority, randomized controlled clinical trial is going to be designed to assess efficacy for low-risk patients, identified with pretreatment plasma EBV DNA <4000 copies/mL, received 2 cycle cisplatin-based chemotherapy compared with 3 cycle cisplatin-based chemotherapy. Therefore, combining the plasma EBV DNA might guide individualized concurrent chemoradiotherapy for advanced NPC patients in the future.

The major limitation of our study is that a single measurement of EBV DNA and the data were obtained exclusively at 1 center, and the measurement of plasma EBV DNA still needs to be globally standardized. The second limitation is that was that the time of follow-up was shorter in this study, and close monitoring and 5-year follow-up data are still required for these patients. The third shortcoming is that we still lacked of post-treatment EBV DNA level, future study need to continue to evaluate the prognostic value of post-treatment EBV DNA in the IMRT value.

In summary, elevated plasma EBV DNA was still an effective prognostic biomarker for local and regionally advanced NPC patients in the IMRT area. Future ramdomized clinical trials are needed to further evaluate whether plasma EBV DNA levels could be applied to guide individualized treatment for locoregionally advanced stage NPC patients.
